# Performance of Two‐Tiered Subclassification of Atypia of Undetermined Significance in Thyroid Fine‐Needle Aspiration Without Routine Molecular Testing

**DOI:** 10.1002/dc.70034

**Published:** 2025-10-23

**Authors:** Pocholo D. Santos, Chiung‐Ru Lai, Jen‐Fan Hang

**Affiliations:** ^1^ Department of Pathology and Laboratories Makati Medical Center Makati Philippines; ^2^ Department of Pathology and Laboratory Medicine Taipei Veterans General Hospital Taipei Taiwan; ^3^ Department of Pathology, School of Medicine National Yang Ming Chiao Tung University Taipei Taiwan; ^4^ Institute of Clinical Medicine, National Yang Ming Chiao Tung University Taipei Taiwan

**Keywords:** atypia of undetermined significance (AUS), molecular testing, risk of malignancy (ROM), risk of neoplasm (RON), the bethesda system for reporting thyroid cytopathology (TBSRTC), thyroid fine‐needle aspiration (FNA)

## Abstract

**Background:**

The third edition of the Bethesda system for reporting thyroid cytopathology recommends a simplified two‐tiered subclassification of atypia of undetermined significance (AUS), dividing cases into AUS with nuclear atypia (AUS‐Nuclear) and other atypia (AUS‐Other). This study aims to evaluate the performance of these subcategories in estimating the risk of malignancy (ROM) in a setting without routine molecular testing.

**Methods:**

A retrospective review was conducted on consecutive thyroid fine‐needle aspiration (FNA) cases diagnosed as AUS between 2018 and 2023. Surgical pathology reports were matched with the FNA‐targeted nodules to enable cyto‐histologic correlation. ROM and risk of neoplasm (RON) were calculated for all AUS cases and for each subcategory.

**Results:**

Among 16,030 thyroid FNA cases, 617 (3.8%) were diagnosed as AUS. Histologic follow‐up was available in 190 cases. Final diagnoses included non‐neoplastic lesions (42.6%), benign neoplasms (17.4%), low‐risk neoplasms (5.3%), and malignant neoplasms (34.7%). The ROM for AUS‐Nuclear was significantly higher at 54.7%, compared to 14.7% for AUS‐Other (*p* < 0.001). Similarly, the RON was significantly higher in the AUS‐Nuclear group (67.4%) than in the AUS‐Other group (47.4%) (*p* = 0.005).

**Conclusion:**

AUS‐Nuclear carries a substantially higher ROM than AUS‐Other, with a ROM (54.7%) comparable to the reported positive predictive values of molecular assays such as Afirma GSC (47%, 95% CI: 36%–58%) and ThyroSeq v3 (66%, 95% CI: 56%–75%). These findings support the clinical utility of the two‐tiered AUS subclassification in enhancing risk stratification, particularly in settings where molecular testing is not routinely available.

## 1 Introduction

The Bethesda system for reporting thyroid cytopathology (TBSRTC) has standardized the classification of thyroid cytology, offering a structured framework to facilitate consistent diagnostic practices and risk stratification. However, indeterminate categories, particularly atypia of undetermined significance (AUS) and follicular neoplasm (FN), remain areas of diagnostic uncertainty. The third edition of TBSRTC introduced significant updates, one of the most important being the two‐tiered subclassification for AUS, aimed at refining risk stratification and guiding management [1]. This subclassification delineates AUS cases based on the presence of nuclear atypia, which has been shown to correlate with a higher risk of malignancy (ROM) [2, 3, 4, 5, 6, 7, 8]. Based on the current classification, AUS is recommended to be categorized into “nuclear atypia” (previously referred to as “cytologic atypia” in the second edition) and “other” [1, 9]. There is emphasis on recognizing different types of nuclear atypia, including focal nuclear atypia, diffuse mild nuclear atypia, a combination of nuclear and architectural atypia, atypical cyst‐lining cells, and atypical histiocytoid cells [10]. These patterns are prioritized over other morphologic patterns such as architectural, oncocytic, and lymphocytic atypia, which are generally associated with a lower ROM.

The indeterminate nature of AUS causes clinical uncertainty, with management options ranging from observation and repeat fine‐needle aspiration (FNA) to surgical excision. In recent years, molecular testing has emerged as a pivotal tool in the evaluation of AUS, endorsed by the American Thyroid Association (ATA) guidelines [11]. Molecular assays help refine risk stratification by identifying genetic alterations associated with malignancy, thus aiding in clinical decision‐making. This approach has gained widespread acceptance in the United States, where major insurance providers typically cover the cost. The afirma genomic sequencing classifier (GSC) and Thyroseq v3 are some of the examples that are commonly used [12, 13]. The application of molecular testing in indeterminate thyroid cytology has also been adopted in select regions of Europe and Canada [14, 15]. Previous studies have demonstrated differential risks when molecular testing is combined with AUS subclassification [16, 17, 18, 19, 20, 21]. However, the adoption of molecular testing in Asia remains limited, owing to factors such as cost, lack of reimbursement, and variable access to advanced diagnostic infrastructure. This disparity highlights the need for further evaluation of alternative approaches to AUS risk stratification in resource‐limited settings. This study aims to explore whether binary AUS subcategories can provide a reliable and comparable risk stratification without the adjunct of molecular testing in a large institutional cohort, thereby assessing its standalone diagnostic value in clinical decision‐making.

## 2 Materials and Methods

### 2.1 Case Selection and Cyto‐Histologic Correlation

This study was approved by the institutional review board of Taipei Veterans General Hospital, Taipei, Taiwan (IRB no.: 2025‐04‐010 AC). A retrospective review of pathology archives was conducted to identify patients diagnosed with AUS on thyroid FNA from 2018 to 2023, with corresponding thyroidectomy specimens available. The targeted nodule from ultrasound‐guided FNA was carefully correlated with the gross description in the final pathology report. Incidental microcarcinomas not corresponding to the targeted FNA lesion were excluded from cyto‐histologic correlation.

Cytopathology reports were reviewed and all AUS cases were subcategorized based on the second edition of TBSRTC into cytologic atypia (AUS‐C), architectural atypia (AUS‐A), cytologic and architectural atypia (AUS‐C/A), Hürthle cell aspirates (AUS‐H), and not otherwise specified (AUS‐NOS). All cases were then reclassified based on the third edition of TBSRTC. AUS cases with nuclear atypia (AUS‐Nuclear) were clustered from AUS‐C and AUS‐C/A, while the remaining cases were categorized as AUS‐Other. The corresponding histologic diagnosis was categorized into four groups: non‐neoplastic, benign neoplasm, low‐risk neoplasm, and malignant based on the fifth edition of the World Health Organization classification of endocrine and neuroendocrine tumors [22].

### 2.2 Statistical Analysis

Resection rate, ROM, and risk of neoplasm (RON) were analyzed for each AUS subcategory, and comparisons were made between the second and third editions of TBSRTC. The chi‐square test was used for categorical variable comparisons, with statistical significance defined as a two‐tailed *p*‐value < 0.05. Data analysis was performed using Social Science Statistics chi‐square test calculator [23].

## 3 Results

### 3.1 Clinical and Cytologic Characteristics

**FIGURE 1:**
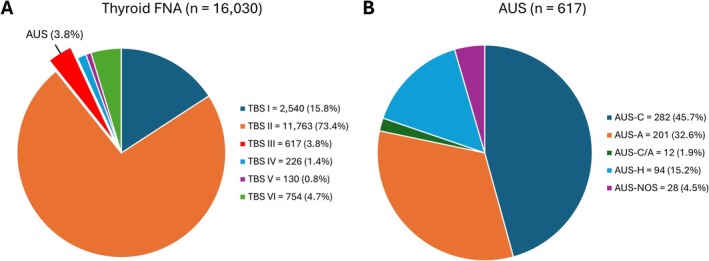
Distribution of thyroid fine needle aspiration (FNA) and atypia of undetermined significance (AUS) cases from 2018 to 2023. (A) Total FNA cases categorized according to the Bethesda system (TBS). (B) Subclassification of AUS cases based on the second edition of TBS. [Color figure can be viewed at wileyonlinelibrary.com]

**FIGURE 2:**
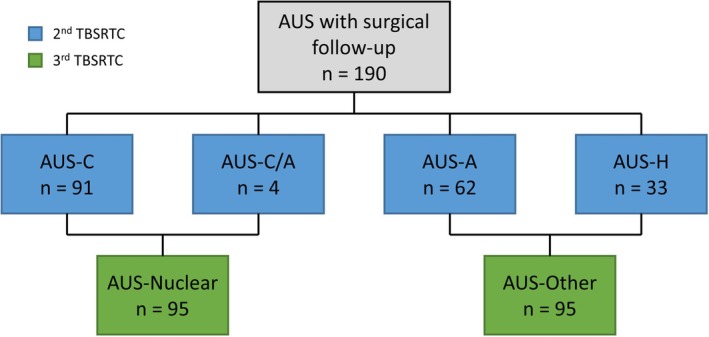
Classification of atypia of undetermined significance (AUS) cases with surgical follow‐up based on the second and third editions of the Bethesda system for reporting thyroid cytopathology (TBSRTC). [Color figure can be viewed at wileyonlinelibrary.com]

A total of 16,030 FNAs were identified during the study period, including 2540 (15.8%) cases as non‐diagnostic, 11,763 (73.4%) as benign, 617 (3.8%) as AUS, 226 (1.4%) as FN, 130 (0.8%) as suspicious for malignancy, and 754 (4.7%) as malignant (Figure 1A). The distribution of AUS subcategories based on the second edition of TBSRTC was as follows: 282 (45.7%) cases as AUS‐C, 201 (32.6%) cases as AUS‐A, 12 (2%) cases as AUS‐C/A, 94 (15.2%) cases as AUS‐H, and 28 (4.5%) cases as AUS‐NOS (Figure 1B). Among the AUS FNAs, 197 had corresponding thyroidectomy specimens. Seven patients underwent two repeated FNAs with the same AUS subcategory results. The study cohort comprised 190 patients, including 139 females, and 51 males, with a mean age of 52 years (range: 20–86 years). Notably, no AUS‐NOS cases proceeded to surgery. The distribution of AUS subcategories within the study cohort, according to both the second and third editions of TBSRTC, is summarized in Figure 2.

### 3.2 Cyto‐Histologic Correlation

**FIGURE 3:**
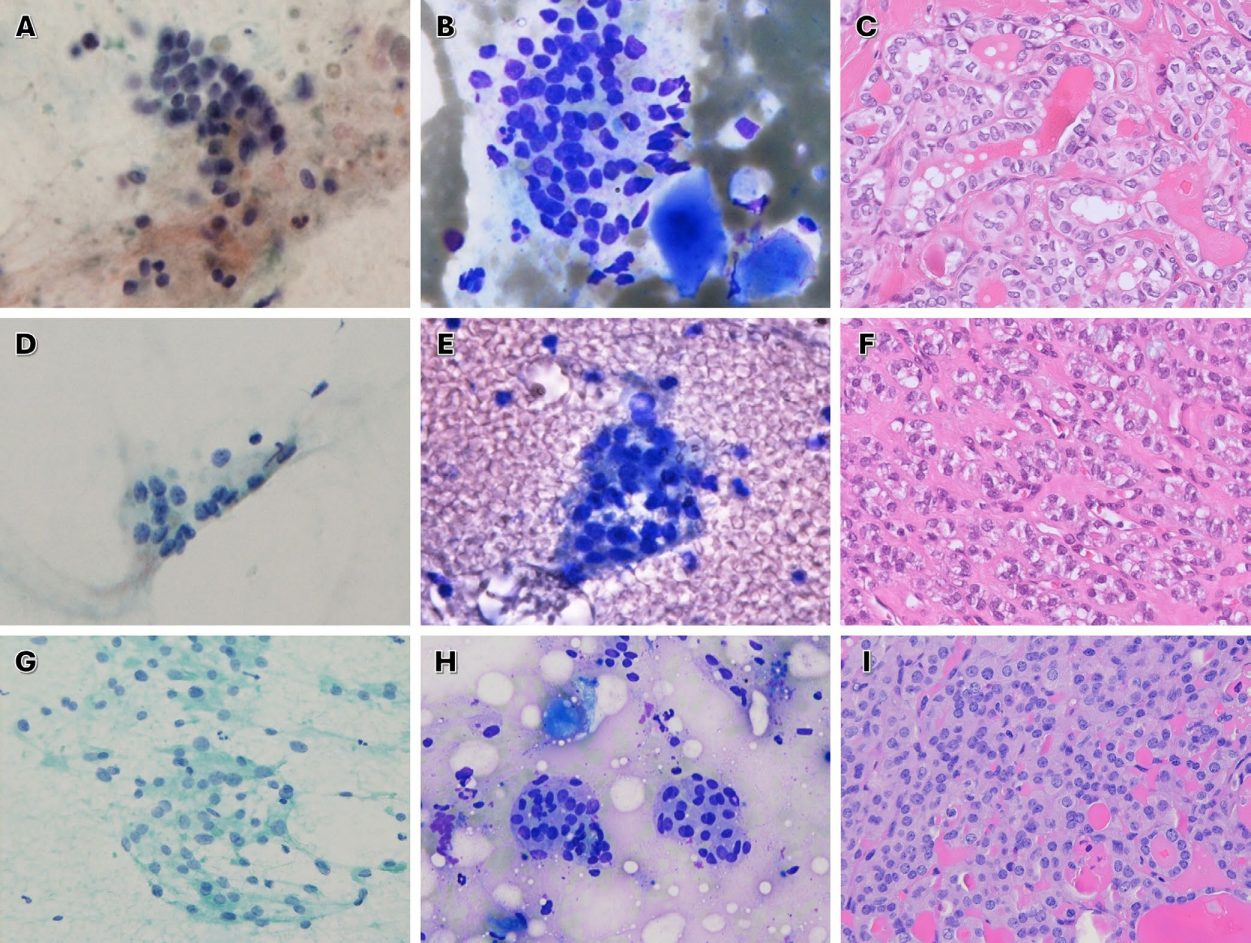
Cyto‐histologic correlation across atypia of undetermined significance (AUS) subcategories. A case of AUS‐C: (A) Pap‐stained SurePath preparation showing scant clusters of follicular cells with mild nuclear elongation, clearing, and overlapping arrangement (800×). (B) Dry smear with Liu stain demonstrating similar cytomorphology and scattered bubble gum–type dense colloid (800×). (C) Corresponding thyroidectomy specimen showing a classic papillary microcarcinoma (H&E, 400×). A case of AUS‐A: (D) Pap‐stained SurePath preparation showing very scant follicular cells with slightly enlarged nuclei with *RAS*‐like nuclear change in a vague microfollicular pattern (800×). (E) Dry smear with Liu stain showing similar cytomorphology on a bloody background without colloid (800×). (F) Thyroidectomy specimen revealing a NIFTP (H&E, 400×). A case of AUS‐H: (G) Pap‐stained direct smear showing oncocytic cell fragments with anisonucleosis and dense granular cytoplasm (400×). (H) Dry smear with Liu stain demonstrating similar cytomorphology with minimal background colloid (400×). (I) Thyroidectomy specimen showing an oncocytic adenoma (H&E, 400×). [Color figure can be viewed at wileyonlinelibrary.com]

The detailed cyto‐histologic correlation utilizing the second edition of TBSRTC is summarized in Table 1. In the AUS‐C subcategory (Figure 3A,B), malignant neoplasms were the most frequent histologic outcome, accounting for 54.9% of cases. Among these, the vast majority (92%) were papillary thyroid carcinomas (PTCs) (Figure 3C). Non‐neoplastic disease represented the second most common outcome (31.9%), predominantly follicular nodular disease (FND, 82.8%). Benign neoplasms and low‐risk neoplasms accounted for 9.9% and 3.3%, respectively. Only three cases were classified as non‐invasive follicular tumor with papillary like features (NIFTP). In the AUS‐A subcategory (Figure 3D,E), non‐neoplastic disease was the most common diagnosis (51.6%), with FND comprising 93.8% of these cases. This was followed by benign neoplasm (25.8%), primarily follicular adenomas (81.3%). Malignant neoplasms were identified in 14.5% of cases, including four PTCs and four follicular thyroid carcinomas (FTCs). Low‐risk neoplasms accounted in 8.1%, composed of three NIFTP (60%) (Figure 3F) and two follicular tumors of uncertain malignant potential (FTUMP, 40%). For AUS‐H subcategory (Figure 3G,H), the most frequent outcome was non‐neoplastic disease (51.6%), with FND representing 88.9% of these cases. Benign neoplasms accounted for 24.2%, most commonly oncocytic adenoma (87.5%) (Figure 3I). Malignant neoplasms were present in 15.2%, predominantly PTC (60%). Low‐risk neoplasm were seen in 6.1%, both diagnosed as oncocytic tumor of uncertain malignant potential (OTUMP). Only four cases were subcategorized as AUS‐C/A. Among these, two were PTCs and two were FND.

**TABLE 1:** Histologic correlation of the atypia of undetermined significance (AUS) subcategories (TBSRTC, 2nd ed.).

Final histologic diagnosis	AUS‐C (*n* = 91, 47.9%)	AUS‐A (*n* = 62, 32.6%)	AUS‐H (*n* = 33, 17.4%)	AUS‐C/A (*n* = 4, 2.1%)	Total (*n* = 190, 100%)
Non‐neoplastic disease	29 (31.9%)	32 (51.6%)	18 (54.5%)	2 (50%)	81 (42.6%)
Follicular nodular disease	24 (82.8%)	30 (93.8%)	16 (88.9%)	2 (100%)	72 (88.9%)
Chronic lymphocytic thyroiditis	5 (17.2%)	1 (3.1%)	1 (5.6%)	0 (0%)	7 (8.6%)
Diffuse hyperplasia	0 (0%)	0 (0%)	1 (5.6%)	0 (0%)	1 (1.2%)
Granulomatous thyroiditis	0 (0%)	1 (3.1%)	0 (0%)	0 (0%)	1 (1.2%)
Benign neoplasm	9 (9.9%)	16 (25.8%)	8 (24.2%)	0 (0%)	33 (17.4%)
Follicular adenoma	6 (66.7%)	13 (81.3%)	1 (12.5%)	0 (0%)	20 (60.6%)
Oncocytic adenoma	3 (33.3%)	3 (18.8%)	7 (87.5%)	0 (0%)	13 (39.4%)
Low risk neoplasm	3 (3.3%)	5 (8.1%)	2 (6.1%)	0 (0%)	10 (5.3%)
NIFTP	3 (100%)	3 (60%)	0 (0%)	0 (0%)	6 (60%)
FTUMP	0 (0%)	2 (40%)	0 (0%)	0 (0%)	2 (20%)
OTUMP	0 (0%)	0 (0%)	2 (100%)	0 (0%)	2 (20%)
Malignant neoplasm	50 (54.9%)	9 (14.5%)	5 (15.2%)	2 (50%)	66 (34.7%)
Papillary thyroid carcinoma	46 (92%)	4 (44.4%)	3 (60%)	2 (100%)	55 (83.3%)
Classical	42 (84%)	1 (11.1%)	2 (40%)	2 (100%)	47 (71.2%)
Tall cell	2 (4%)	0 (0%)	0 (0%)	0 (0%)	2 (3%)
Infiltrating follicular	1 (2%)	1 (11.1%)	0 (0%)	0 (0%)	2 (3%)
Oncocytic	0 (0%)	1 (11.1%)	1 (20%)	0 (0%)	2 (3%)
Invasive encapsulated follicular	0 (0%)	1 (11.1%)	0 (0%)	0 (0%)	1 (1.5%)
Diffuse sclerosing	1 (2%)	0 (0%)	0 (0%)	0 (0%)	1 (1.5%)
Follicular thyroid carcinoma	1 (2%)	4 (44.4%)	0 (0%)	0 (0%)	5 (7.6%)
Oncocytic thyroid carcinoma	0 (0%)	0 (0%)	1 (20%)	0 (0%)	1 (1.5%)
Medullary thyroid carcinoma	1 (2%)	1 (11.1%)	1 (20%)	0 (0%)	3 (4.5%)
Anaplastic thyroid carcinoma	1 (2%)	0 (0%)	0 (0%)	0 (0%)	1 (1.5%)
Metastatic carcinoma	1 (2%)	0 (0%)	0 (0%)	0 (0%)	1 (1.5%)

Abbreviations: FTUMP, follicular tumor of uncertain malignant potential; NIFTP, non‐invasive follicular tumor with papillary like features; OTUMP, oncocytic tumor of uncertain malignant potential.

The histologic correlation based on the third edition of TBSRTC is summarized in Table 2, with an equal number of cases in both the AUS‐Nuclear and AUS‐Other subcategories (*n* = 95 each). In the AUS‐Nuclear subcategory, the most common outcome was malignant neoplasm (54.7%), with PTC accounting for 92.3% of these cases. This was followed by non‐neoplastic disease (32.6%), mostly FND (83.9%). Benign neoplasms accounted for 9.5% of cases, most commonly follicular adenoma (66.7%). Low‐risk neoplasms were identified in 3.2%, all of which were NIFTP. In contrast, AUS‐Other cases were most commonly associated with non‐neoplastic disease (52.6%), with FND representing 92% of these cases. Benign neoplasms accounted for 25.3%, most commonly follicular adenoma (58.3%). Malignant neoplasms comprised 14.7% of cases, including PTC (50%) and FTC (28.6%) as the most frequent diagnoses. Lastly, low‐risk neoplasms were identified in 7.4% of cases, with NIFTP representing the most common (42.9%).

**TABLE 2:** Histologic correlation of the atypia of undetermined significance (AUS) subcategories (TBSRTC, 3rd ed.).

Final histologic diagnosis	AUS‐Nuclear (*n* = 95, 50%)	AUS‐Other (*n* = 95, 50%)	Total (*n* = 190, 100%)
Non‐neoplastic disease	31 (32.6%)	50 (52.6%)	81 (42.6%)
Follicular nodular disease	26 (83.9%)	46 (92%)	72 (88.9%)
Chronic lymphocytic thyroiditis	5 (16.1%)	2 (4%)	7 (8.6%)
Diffuse hyperplasia	0 (0%)	1 (2%)	1 (1.2%)
Granulomatous thyroiditis	0 (0%)	1 (2%)	1 (1.2%)
Benign neoplasm	9 (9.5%)	24 (25.3%)	33 (17.4%)
Follicular adenoma	6 (66.7%)	14 (58.3%)	20 (60.6%)
Oncocytic adenoma	3 (33.3%)	10 (41.7%)	13 (39.4%)
Low risk neoplasm	3 (3.2%)	7 (7.4%)	10 (5.3%)
NIFTP	3 (100%)	3 (42.9%)	6 (60%)
FTUMP	0 (0%)	2 (28.6%)	2 (20%)
OTUMP	0 (0%)	2 (28.6%)	2 (20%)
Malignant neoplasm	52 (54.7%)	14 (14.7%)	66 (34.7%)
Papillary thyroid carcinoma	48 (92.3%)	7 (50%)	55 (83.3%)
Classical	44 (84.6%)	3 (21.4%)	47 (71.2%)
Tall cell	2 (3.8%)	0 (0%)	2 (3%)
Infiltrating follicular	1 (1.9%)	1 (7.1%)	2 (3%)
Oncocytic	0 (0%)	2 (14.3%)	2 (3%)
Invasive encapsulated follicular	0 (0%)	1 (7.1%)	1 (1.5%)
Diffuse sclerosing	1 (1.9%)	0 (0%)	1 (1.5%)
Follicular thyroid carcinoma	1 (1.9%)	4 (28.6%)	5 (7.6%)
Oncocytic thyroid carcinoma	0 (0%)	1 (7.1%)	1 (1.5%)
Medullary thyroid carcinoma	1 (1.9%)	2 (14.3%)	3 (4.5%)
Anaplastic thyroid carcinoma	1 (1.9%)	0 (0%)	1 (1.5%)
Metastatic carcinoma	1 (1.9%)	0 (0%)	1 (1.5%)

Abbreviations: FTUMP, follicular tumor of uncertain malignant potential; NIFTP, non‐invasive follicular tumor with papillary like features; OTUMP, oncocytic tumor of uncertain malignant potential.

### 3.3 Resection Rate, ROM, and RON of AUS Subcategories

Table 3 summarizes the resection rate, ROM, and RON across AUS subcategories according to the second edition of TBSRTC. The overall resection rate was 30.8%, with no significant difference among the subcategories (*p* = 0.910). However, there were significant differences in ROM and RON. The ROM was 54.9% for AUS‐C, 50.0% for AUS‐C/A, 14.5% for AUS‐A, and 15.2% for AUS‐H, respectively (*p* < 0.001). The RON was 68.1% for AUS‐C, 50.0% for AUS‐C/A, 48.4% for AUS‐A, and 45.5% for AUS‐H, respectively (*p* = 0.039). None of the AUS‐NOS cases underwent resection.

**TABLE 3:** Risk of malignancy (ROM) and risk of neoplasm (RON) in different atypia of undetermined significance (AUS) subcategories (TBSRTC 2nd ed.).

AUS subcategories	Total	Resection rate (%)	*p*	Malignant	ROM	*p*	Neoplasm	RON	*p*
AUS‐C	282	91 (32.3%)	0.910	50	54.9%	**< 0.001**	62	68.1%	**0.039**
AUS‐C/A	12	4 (33.3%)		2	50%		2	50%	
AUS‐A	201	62 (30.8%)		9	14.5%		30	48.4%	
AUS‐H	94	33 (35.1%)		5	15.2%		15	45.5%	
AUS‐NOS	28	0 (0%)		n/a	n/a		n/a	n/a	
Total	617	190 (30.8%)		66	34.7%		109	57.4%	

*Note:* Bold font indicates statistical significance.

Table 4 summarizes the resection rate, ROM, and RON across AUS subcategories according to the third edition of TBSRTC. The overall resection rate did not show a significant difference between AUS‐Nuclear and AUS‐Other (32.3% vs. 29.4%, *p* = 0.436). However, there were significant differences in ROM and RON. The ROM was 54.7% for AUS‐Nuclear and 14.7% for AUS‐Other, respectively (*p* < 0.001). The RON was 67.4% for AUS‐Nuclear and 47.4% for AUS‐Other, respectively (*p* = 0.005).

**TABLE 4:** Risk of malignancy (ROM) and risk of neoplasm (RON) in different atypia of undetermined significance (AUS) subcategories (TBSRTC 3rd ed.).

AUS subcategories	Total	Resection rate (%)	*p*	Malignant	ROM	*p*	Neoplasm	RON	*p*
AUS‐Nuclear	294	95 (32.3%)	0.436	52	54.7%	**< 0.001**	64	67.4%	**0.005**
AUS‐Other	323	95 (29.4%)		14	14.7%		45	47.4%	
Total	617	190 (30.8%)		66	34.7%		109	57.4%	

*Note:* Bold font indicates statistical significance.

## 4 Discussion

Thyroid FNA is a widely used and effective diagnostic tool for evaluating thyroid nodules, with TBSRTC providing standardized terminology and estimated ROM for each diagnostic category [1]. Among these, the category of AUS remains particularly challenging due to its broad cytologic heterogeneity and variable malignancy risk, underscoring the need for more refined risk stratification strategies [24, 25, 26]. The frequency and interpretation of AUS diagnoses can vary significantly among institutions, influenced by differences in cytopathologic thresholds, patient populations, referral patterns, and interobserver variability in assessing subtle nuclear and architectural atypia [27, 28]. While TBSRTC recommends AUS rates of less than 7%–10% with an associated ROM of 10%–30% [1, 9], real‐world data demonstrate a much broader range. Reported AUS rates in the literature vary from as low as 1% to as high as 22.6% [29, 30, 31], and the ROM with surgical follow‐up has been reported as high as 68.6% [10], reflecting the practical complexities in applying this diagnostic category consistently.

In our study, the overall AUS rate was relatively low at 3.8%, likely reflecting the application of stricter diagnostic thresholds and institutional practices that enhance specificity. One contributing factor may be our relatively high non‐diagnostic rate of 15.8%, near the upper limit of the recommended range, which is likely due to the lack of routine on‐site evaluation. The inverse relationship between non‐diagnostic and AUS rates is well established, suggesting that cases with borderline features may be classified as non‐diagnostic rather than AUS based on adequacy criteria or interpretive approach [32]. Additionally, as a high‐volume referral center, our institution also encounters a greater proportion of complex, high‐risk thyroid nodules that may be more appropriately classified into higher‐risk categories such as suspicious for malignancy or malignant, rather than AUS. This referral bias elevates the pretest probability of malignancy and may partially account for the higher ROM observed in our AUS cases [33].

In the current study, based on the second edition of TBSRTC, AUS‐C was the most frequently assigned subcategory, followed by AUS‐A, AUS‐H, and AUS‐NOS. The AUS‐C/A subcategory was the least commonly used, likely reflecting our institutional preference for assigning a single dominant atypia pattern rather than combining features. This distribution largely aligns with previously published data [34]. Histologic follow‐up revealed that 34.7% of all AUS cases were malignant, comparable to the upper range of 30% estimated by TBSRTC. AUS‐C and AUS‐C/A demonstrated similarly high ROMs (54.9% and 50%, respectively), whereas AUS‐A and AUS‐H showed much lower ROMs (14.5% and 15.2%, respectively). The distribution of histologic diagnoses also varied across subcategories. PTC was predominantly found in AUS‐C and AUS‐C/A, while follicular and oncocytic neoplasms were more commonly associated with AUS‐A and AUS‐H. These findings further support the simplified two‐tiered subclassification introduced in the third edition of TBSRTC, which groups AUS cases based solely on the presence (AUS‐Nuclear) or absence (AUS‐Other) of nuclear atypia. It is noteworthy that only two cases (2/55) in our AUS cohort represented aggressive histologic subtypes of PTC, both being tall cell PTCs and both initially classified as AUS‐Nuclear. This observation is consistent with the general assumption that aggressive PTC subtypes are seldom assigned to the AUS‐Nuclear category, as their distinctive cytologic features more often warrant a direct malignant diagnosis. Distinguishing invasive encapsulated follicular variant of PTC (*RAS*‐like) from conventional PTC (*BRAF*‐like) is important, as TBSRTC recommends assigning *RAS*‐like nuclear atypia to the FN category, whereas *BRAF*‐like atypia is more appropriately classified as suspicious for malignancy or malignant. This principle also underlies our practice of categorizing cases with *RAS*‐like nuclear changes and a vague microfollicular pattern as AUS‐A rather than AUS‐C (Figure 3D–F). In our cohort, the proportion of NIFTP identified among AUS cases was relatively low. This finding may, in part, reflect regional practice patterns, as the diagnostic threshold for recognizing *RAS*‐like nuclear atypia tends to be higher in Asia, leading many NIFTPs to be classified as follicular adenomas in routine practice [35].

Molecular diagnostics, such as Afirma GSC and ThyroSeq v3, have significantly influenced the risk stratification of thyroid nodules categorized as AUS in the United States and other Western countries [12, 13]. These tests provide high negative predictive values, helping to avoid unnecessary surgeries, and moderate positive predictive values, assisting in identifying nodules that warrant surgical intervention. However, concerns have been raised about their actual impact on clinical management. Some studies suggest that molecular testing has had limited influence on surgical decision‐making [36]. In certain cohorts, its introduction has shifted cytologic interpretation toward indeterminate categories, yet the overall rate of thyroidectomy has not declined [37, 38]. Furthermore, meta‐analyzes have shown that molecular testing may lead to increased use of total thyroidectomy and central lymph node dissection, without a corresponding improvement in malignancy detection or reduction in completion thyroidectomy rates [39].

In most Asian countries, multigene panel testing is not routinely incorporated into thyroid FNA practice due to its high cost, lack of reimbursement, and limited accessibility. Nevertheless, studies from these regions have reported lower surgical resection rates and higher risks of malignancy for AUS cases compared to Western countries [40, 41]. In a meta‐analysis by Vuong et al. examining 22 Western series and 16 Asian series, the pooled resection rate was 40.5% for the Western studies and 29.5% for the Asian studies [40]. In our institutional cohort, where molecular testing is not routinely employed, the overall resection rate for AUS was 30.8%. Although we actively promoted AUS subcategorization and regularly shared corresponding ROM data with our clinical colleagues during the study period, resection rates did not differ significantly across AUS subcategories in this retrospective analysis (Tables 3 and 4), suggesting that the potential impact of surgical selection bias on ROM estimates was likely minimal. Subclassification of AUS into AUS‐Nuclear and AUS‐Other yielded significantly different ROM (54.7% vs. 14.7%, *p* < 0.001) and RON (67.4% vs. 47.4%, *p* = 0.005). Notably, the ROM for AUS‐Nuclear closely parallels the reported positive predictive values of advanced molecular assays, such as Afirma GSC (47%, 95% CI: 36%–58%) [42] and ThyroSeq v3 (66%, 95% CI: 56%–75%) [43], as demonstrated in prospective blinded multicenter studies. These findings underscore the high diagnostic value of cytomorphologic subclassification alone. Furthermore, histologic follow‐up provided insight into management strategies. The observed discrepancy between RON and ROM in AUS‐Other suggests that many of these cases represent low‐risk neoplasms, potentially amenable to more conservative management, such as regular follow‐up or simple lobectomy.

A major limitation of this study is that ROM and RON were calculated based on the subset of cases with surgical follow‐up and therefore represent the higher end of the expected range with a potential risk of overestimation. In addition, practice patterns in our setting, such as the lack of routine molecular testing, may contribute to a lower AUS calling rate and different strategies in surgical selection. These factors should be taken into account when interpreting our findings, which are best regarded as reflective of our institutional experience rather than directly generalizable to all practice settings.

In conclusion, our findings demonstrate that cytomorphologic subclassification of AUS into AUS‐Nuclear and AUS‐Other offers meaningful and robust risk stratification. AUS‐Nuclear, in particular, is associated with a significantly higher ROM, approximating the positive predictive values reported for advanced multigene assays. This highlights the potential of careful cytologic evaluation to provide comparable diagnostic insights in the absence of molecular testing. In practice settings where multigene panel testing is limited or unavailable, as is the case in many Asian countries, thorough morphologic subclassification remains a practical and effective strategy for guiding clinical management and surgical decision‐making.

## Author Contributions

**Pocholo D. Santos:** data curation, formal analysis, investigation, and writing – original draft. **Chiung‐Ru Lai:** data curation and investigation. **Jen‐Fan Hang:** conceptualization, data curation, investigation, project administration, supervision, writing – original draft, and writing – review and editing.

## Conflicts of Interest

The authors declare no conflicts of interest.

## Data Availability

The data that support the findings of this study are available on request from the corresponding author. The data are not publicly available due to privacy or ethical restrictions.
